# Cluster Analysis of Short Sensory Profile Data Reveals Sensory-Based Subgroups in Autism Spectrum Disorder

**DOI:** 10.3390/ijms232113030

**Published:** 2022-10-27

**Authors:** Ariel M. Lyons-Warren, Michael F. Wangler, Ying-Wooi Wan

**Affiliations:** 1 Department of Pediatrics, Section of Pediatric Neurology and Developmental Neuroscience, Baylor College of Medicine, 6621 Fannin St., Houston, TX 77030, USA; 2 Jan and Dan Duncan Neurological Research Institute at Texas Children’s Hospital, 1250 Moursund St., Houston, TX 77030, USA; 3 Department of Molecular and Human Genetics, Baylor College of Medicine, Houston, TX 77030, USA

**Keywords:** neurodevelopmental disorders, human genetics, sensory processing, autism spectrum disorder, phenotypic biomarker

## Abstract

Autism spectrum disorder is a common, heterogeneous neurodevelopmental disorder lacking targeted treatments. Additional features include restricted, repetitive patterns of behaviors and differences in sensory processing. We hypothesized that detailed sensory features including modality specific hyper- and hypo-sensitivity could be used to identify clinically recognizable subgroups with unique underlying gene variants. Participants included 378 individuals with a clinical diagnosis of autism spectrum disorder who contributed Short Sensory Profile data assessing the frequency of sensory behaviors and whole genome sequencing results to the Autism Speaks’ MSSNG database. Sensory phenotypes in this cohort were not randomly distributed with 10 patterns describing 43% (162/378) of participants. Cross comparison of two independent cluster analyses on sensory responses identified six distinct sensory-based subgroups. We then characterized subgroups by calculating the percent of patients in each subgroup who had variants with a Combined Annotation Dependent Depletion (CADD) score of 15 or greater in each of 24,896 genes. Each subgroup exhibited a unique pattern of genes with a high frequency of variants. These results support the use of sensory features to identify autism spectrum disorder subgroups with shared genetic variants.

## 1. Introduction

Autism spectrum disorder (ASD) is a neurodevelopmental disorder defined by deficits in social communication and interactions as well as repetitive, restricted behaviors [1]. Despite a high prevalence of at least 1.5% and increasing family and societal costs [2,3,4], there are currently no targeted pharmacological treatments for ASD [5]. A major barrier to development of targeted treatments is the heterogeneity in presentation seen across individuals with ASD [6,7]. Varied clinical presentations likely reflect diverse underlying molecular mechanisms. Similarly, diversity in patient groups may obscure efficacy in treatment trials. Thus, it is necessary to develop a standardized approach to distinguish different subgroups of individuals with ASD based on both clinical features and underlying molecular/genetic etiologies [8,9,10,11].

Sensory processing differences are a common and debilitating component of the diagnostic criteria for ASD [12]. As such, sensory-based subgrouping within ASD is promising in the classification of heterogeneous ASD populations [13]. Notably, sensory profiles where a specific pattern of sensory features is present in a certain clinical population have been applied to other neurodevelopmental disorders such as Angelman, Cornelia de Lange and Fragile X syndromes [14] as well as Phelan-McDermid Syndrome [15,16] and SYNGAP-1 associated intellectual disability [17]. Further, sensory differences have been correlated with other features of ASD such as level of functioning [18,19]. Given the strong evidence supporting the use of sensory features to identify subgroups within ASD, it is not surprising that previous studies have used cluster analysis to identify sensory-based subgroups (reviewed in [20]). The most recent and largest sensory subgrouping study to date used cluster analysis of Short Sensory Profile responses from 599 participants and identified five distinct sensory based clusters [21] which correlated with other behavioral measures. Prior sensory subgrouping in ASD has focused on categories of seeking verses avoiding and levels of sensitivity, which cannot be directly translated to animal models. Further, none of the previously proposed sensory-based subgroups have been linked to underlying molecular mechanisms or genetic variants which are important for translating sensory behaviors into a molecular biomarker. Therefore, the aim of this study was to determine if individuals with ASD could be sub-grouped based on modality specific hyper- and hypo-sensitivity and evaluate if those subgroups could be linked to patterns of associated genes.

Capitalizing on Short Sensory Profile (SSP) and whole genome sequencing (WGS) data from 378 individuals with ASD, collected as part of the Autism Speaks’ MSSNG Database [22], we report six sensory-based ASD subgroups with shared associated genes, paving the way toward better classification of this heterogeneous population using a clinically relevant biomarker that is easily translated to animal models.

## 2. Results

### 2.1. Short Sensory Profile Responses in ASD Are Heterogeneous

**Figure 1:**
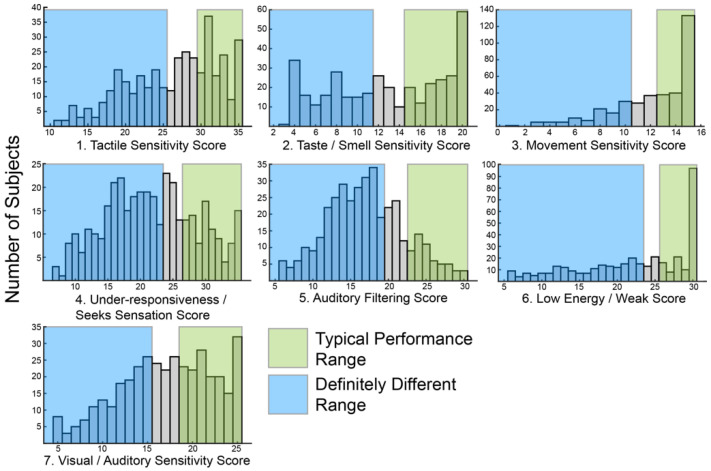
Histograms showing the distribution of raw scores for each of the seven sensory areas defined by the Short Sensory Profile (SSP).

The primary goal of this study was to use sensory features to identify subgroups within ASD. In order to create subgroups, there must be variation within the group. Therefore, we first looked at the distribution of SSP scores within our cohort. Consistent with prior reports [23,24,25,26], a wide range of scores in each of the seven sensory areas assayed by the SSP was observed (Figure 1). Most histograms skewed towards higher values associated with typical scoring ranges. However, the score distributions for under-responsiveness-seeks sensation and auditory filtering exhibited a bell shape distribution with the peak in mid-range scores suggesting most participants were probably different from typically developing children in those areas. Only eight participants (2%) scored in the typically performing range (Figure 1, green shading) for all sensory areas.

Similarly, only 24 participants (6%) scored in the definitely different range (Figure 1, blue shading) for all sensory areas. Scoring in the probably or definitely different range for all sensory areas was slightly more common, seen in 54 participants (14%). Overall, the scores were broadly distributed suggesting sufficient variation to allow for clustering.

Meaningful subgrouping requires that features are not randomly distributed amongst participants. Therefore, we next asked if all possible combinations of sensory features were present in this cohort. If features are random, then all combinations of features should be equally distributed. To test for non-random distribution, data were simplified into binary categories in which all participants were classified as typically performing or different from typical performance including probably different and definitely different using standardized, previously published ranges from a group of 1037 children without disabilities [27]. Using this method, only 82 out of 128 possible combinations of the sensory areas were present in this cohort suggesting that sensory changes are not random and that certain sensory areas tend to be affected together. In fact, the 10 most common patterns described 162/378 participants (43%) (Table 1). 45 patterns were rare, being seen in three or fewer participants each.

**Figure 2:**
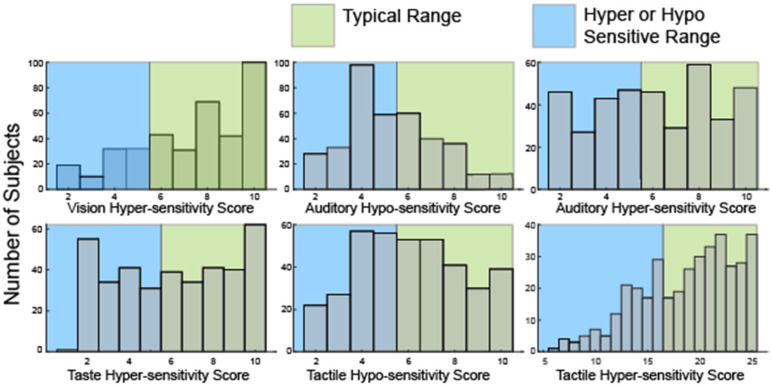
Histograms showing the distribution of raw scores in hyper- or hypo-sensitivity for hearing and touch as well as hyper-sensitivity to vision and taste. No questions in the SSP evaluate hypo-sensitivity to vision or taste or olfactory sensitivities.

Broad heterogeneity was also present in the distribution of hyper—and hypo-sensitivity scores (Figure 2) derived from a subset of the SSP responses (Table 2).

Notably, many participants were both hyper and hyposensitive in the modalities of touch (52, 13.7%) and hearing (111, 29%). Consistent with analysis of the seven sensory areas defined by the SSP, only 35 participants (9%) were hyper-sensitive in all four sensory modalities. 59 participants (15.6%) were neither hyper- nor hypo-sensitive in any sensory modality. Overall, scores in this second analysis were similarly broadly distributed suggesting sufficient variation to allow for clustering.

### 2.2. Cluster Analysis Optimizes at Six Sensory Based Subgroups

**Figure 3:**
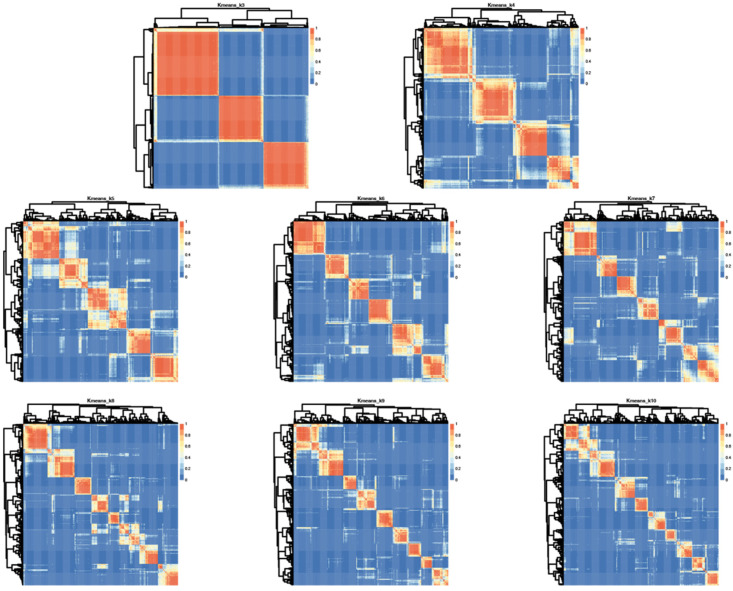
Iterative cluster analysis reveals optimal repeat clustering when using K means = 6. Heatmaps showing overlap for consensus clusters generated using K = 3–10 based on input from all 38 questions in the SSP. Red indicates 100% consensus and blue indicates no consensus. Lighter shading indicates variation across iterations and thus less consensus.

Having determined that SSP scores in this cohort were sufficiently broadly distributed to allow for clustering and that sensory phenotype patterns were not random, we performed cluster analysis on bootstrap samples for 100 iterations using K-means clustering on responses from 377 participants. One participant of the original 378 was excluded due to lack of variance in responses which precludes K-means clustering. The K-mean clustering was run with K = 3 to K = 10 on bootstrap samples for 100 iterations and the consensus clusters for each K were used as the final cluster for subsequent analyses. This consensus cluster analysis was separately performed using both responses to all 38 questions and the hyper/hypo sensitivity subset. Visual inspection of dendrogram based heatmaps generated from consensus clusters (Figure 3) illustrated that K = 3 provided the most consistent clustering.

However, these groupings could be described by “affected in all areas,” “affected in no areas” and “all other participants” which does not provide sufficient clinical granularity to serve as a biomarker. Thus, we also looked at higher K values using analysis of the median distribution. This analysis evaluates consistency of subgroup assignment across the 100 bootstrap iterations and was highest for six subgroups suggesting six groups is optimal for this cohort (Figure S1).

**Figure 4:**
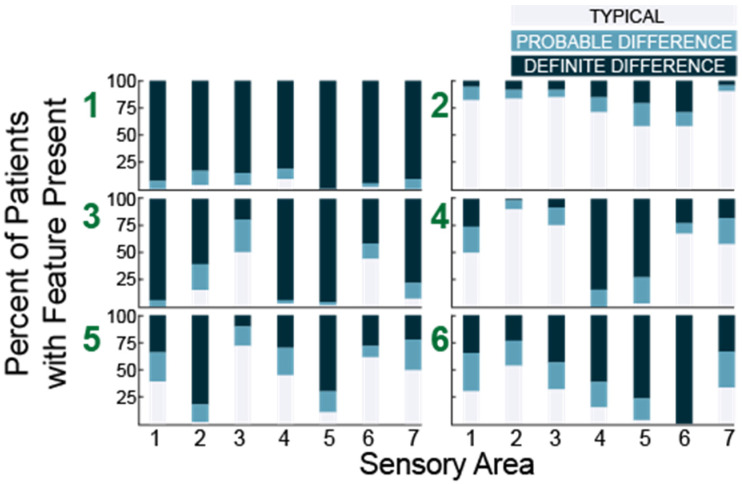
Histograms showing the number of participants with each sensory feature for six subgroups (green numbers) identified in 38 question analysis. Sensory areas (x-axis) are (**1**) Tactile Sensitivity, (**2**) Taste/Smell Sensitivity, (**3**) Movement Sensitivity, (**4**) Underresponsive/Seeks Sensation, (**5**) Auditory Filtering, (**6**) Low Energy/Weak, and (**7**) Visual/Auditory Sensitivity.

Using histograms, we observed that group 1 (*N* = 53) was characterized by high percentage of participants who scored probably or definitely different in all seven areas while group 2 (*N* = 72) was characterized by predominantly typical performance in all areas. Each of the remaining four subgroups (in order, *N* = 63, 62, 67 and 60) exhibited a unique pattern (Figure 4).

**Figure 5:**
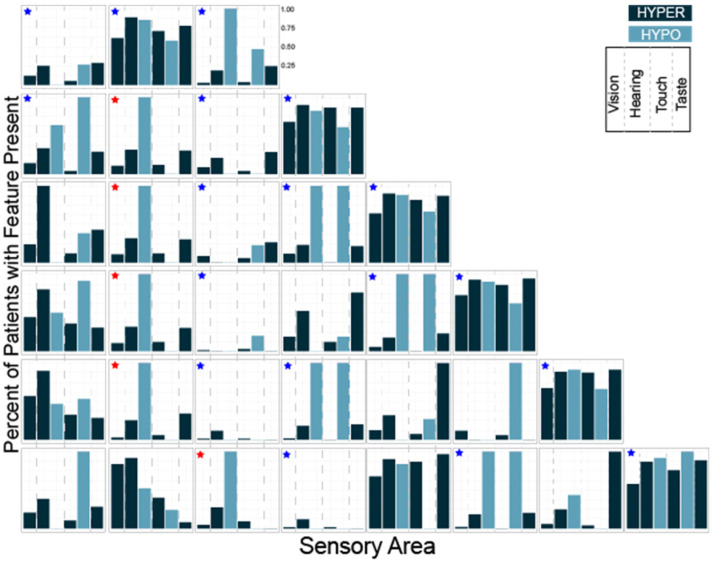
Histograms showing the number of participants with each sensory feature for K = 3 through K = 8 clustering. The three groups present regardless of the total number of clusters are marked with blue stars. Groups characterized by differences in hearing and taste or isolated auditory hyposensitivity are marked with red stars.

Next, we performed the same analysis for K = 3–10 using the subset of questions evaluating hyper- and hypo- sensitivity (Table 2). Looking at the distributions of hyper- and hypo-sensitivity we noticed that K = 3 in this analysis generated a group that was atypical, a group that was typical and a group characterized primarily by differences in hearing and touch. Notably, these three groups were present regardless of the total number of clusters (Figure 5, blue stars). We also noted that a group characterized by differences in hearing and taste and a group characterized by isolated auditory changes were seen repeatedly with different numbers of clusters (Figure 5, red stars). Finally, we observed that at K = 8, groups began to emerge which were atypical in all areas except one, suggesting eight or more clusters would overfit the data.

Thus, both cluster analyses identified a cohort of participants that were very affected, a group mostly scoring in the typically developing or unaffected range and at least three unique clusters with distinct patterns of sensory features.

**Figure 6:**
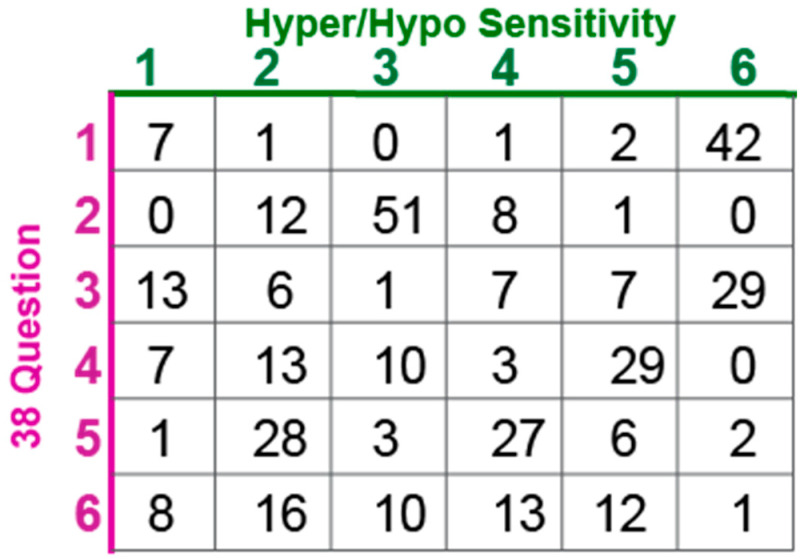
Participant overlap between clustering methods for K = 6.

Having identified phenotypically distinct subgroups using both cluster analyses, we next used the overlap between the two results to better characterize each subgroup. The overlap between the two methods allowed us to clarify the characteristics associated with each cluster which were used to name the clusters (Figure 6). We performed the overlap comparison between the 6 clusters identified in the 38Question analysis and the subgroups identified for K = 5 (Rand index = 0.76), 6 (Rand index = 0.79) and 7 (Rand index = 0.8) of the Hyper/hypo sensitivity analysis. The results were remarkably similar as would be expected given the consistent presence of the same types of subgroups (Figure 5). For simplicity, we present the comparison to Hyper/hypo sensitivity analysis K = 6.

Participants assigned to group 1 in the 38Question analysis strongly overlapped (42/53) with group 6 from the Hyper/hypo sensitivity analysis consistent with both of these subgroups exhibiting differences in all sensory areas and thus we call this subgroup “Atypical in all areas.” Participants assigned to group 2 in the 38Question analysis strongly overlapped (51/72) with group 3 from the Hyper/hypo sensitivity analysis consistent with both of these subgroups exhibiting typical performance in all sensory areas and thus we call this subgroup “Typical in all areas.”

Participants assigned to group 3 in the 38Question analysis overlapped best with group 6 (29/63) and group 1 (13/63) from the Hyper/hypo sensitivity analysis. This group was characterized by differences in tactile sensitivity, auditory filtering, and underresponsive/seeking on the 38Question analysis which was consistent with differences in both hyper- and hypo- sensitivity for tactile and auditory processing. Therefore, we call this subgroup “Tactile and Auditory.” Participants assigned to group 4 in the 38Question analysis overlapped best with Hyper/hypo sensitivity analysis group 5 (29/62). Unlike the prior group, these participants were uniquely hypo-sensitive in the areas of touch and hearing as evidenced by the Hyper/hypo sensitivity analysis and underresponsive/seeking on the 38Question analysis. Therefore, we call this subgroup “Tactile and Auditory Hyposensitive.”

Participants assigned to group 5 in the 38Question analysis overlapped best with group 2 (28/67) and group 4 (27/67) on the Hyper/hypo analysis. This combination was particularly interesting to us because group 5 in the 38Question analysis was defined by differences in taste and auditory sensitivity whereas groups 2 and 4 in the Hyper/hypo analysis were defined by auditory hypo-sensitivity and taste hyper-sensitivity respectively. Thus, we call this group “Taste and Auditory.” Finally, participants assigned to group 6 in the 38Question analysis were mixed, consistent with no strong overlap with any one group from the Hyper/hypo analysis. Therefore, we call this group “Mixed.” Thus, cluster analysis identified a cohort of participants likely to be different in all or almost all areas, participants mostly scoring in the typically developing range, three unique clusters with distinct patterns of sensory features, and one mixed cluster that was not well characterized. There were significantly fewer males in the Taste and Auditory group (subgroup 5) compared to the other groups (Wilcoxon rank-sum test, *p* < 0.02). There was no difference in mean age, adaptive behavior score, socialization score or full-scale IQ between subgroups (Table 3). Thus, we concluded that sensory features can reliably be used to generate clinically distinct subgroups within a heterogeneous population of individuals with ASD. Further, we conclude that in the MSSNG cohort, there are six distinct subgroups.

### 2.3. Subgroups Are Characterized by Unique Patterns of Genetic Variants

Having successfully identified six sensory based subgroups, we next used whole genome sequencing to ask which genes were most likely to have variants for each subgroup. The analysis included variants from the MSSNG database with a Combined Annotation Dependent Depletion (CADD) score of at least 15 and annotation to a gene. These variants were aggregated to a gene level for each participant for further analysis [28]. Whole genome sequencing results were not available for eighteen of the participants including some from each subgroup. In total, we identified 24,896 genes for which at least one participant had an annotated variant.

**Figure 7:**
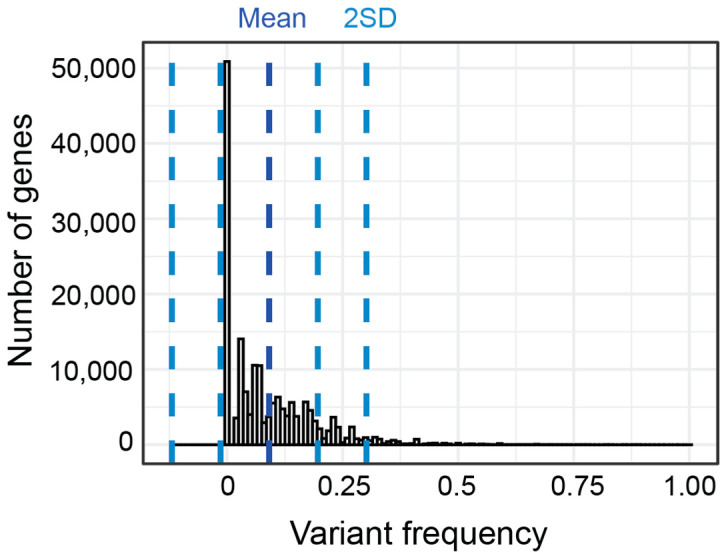
Distribution and standard deviation (blue lines) of GVF for all genes for all subgroups.

As an exploratory analysis, we first calculated the percent of patients in each subgroup who had variants with a CADD score of 15 or greater in each gene. By collapsing across variants within genes, we aimed to be hypothesis generating rather than demonstrating variant specific correlations. We use the term gene variant frequency (GVF) to describe the frequency of patients with variants in a given gene. The mean GVF for all 24,896 genes for all patients was 8.44% (Figure 7). Most genes had a low GVF, with the mean GVF at 2SD being only 27%. Many (51.2%) genes had a low GVF in all subgroups meaning less than 10% of participants in each subgroup had a variant. In fact, the mean GVF for each subgroup was less than 10% (range 7.0–9.9%). The ‘Atypical in all areas’ subgroup had the highest mean GVF at 9.9% although this was not statistically different (*p* > 0.05, Kruskal–Wallis).

**Figure 8:**
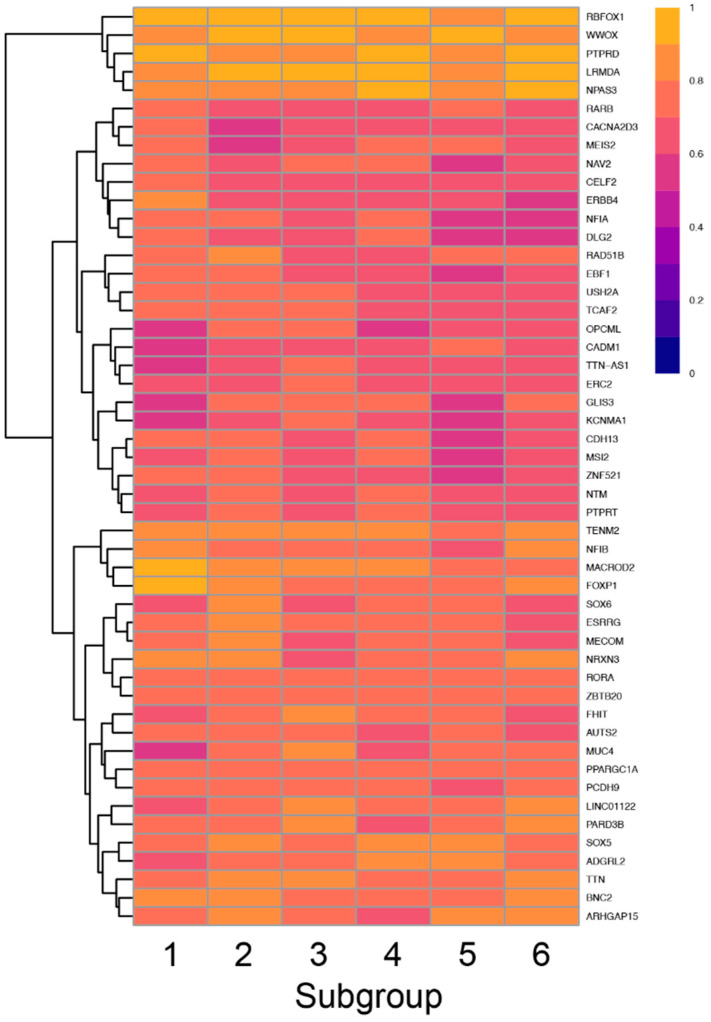
Heatmap showing the frequency of variants in each subgroup (columns) for the top 50 genes (rows) with the highest GVF demonstrating the variation in GVF across subgroups.

To screen for enrichment of variants in a given subgroup, we first asked which genes in each subgroup had the highest GVF. Only 73 genes had an average GVF of 60% or higher with the top 5 genes having variants in 84% or greater of all participants in each group (Figure 8).

Thus, there was significant overlap in the top 10 genes for each subgroup, although the specific pattern of genes was unique for each subgroup. Interestingly, four of the six subgroups had at least one unique gene including FOXP1 which was uniquely enriched in the Atypical in all Areas subgroup, SOX6 and NRXN3 which were uniquely enriched in the Typical in all Areas subgroup, MUC4 and PARD3B which were uniquely enriched in the Tactile and Auditory subgroup and ARHGAP15 which was uniquely enriched in the Taste and Auditory subgroup.

**Figure 9:**
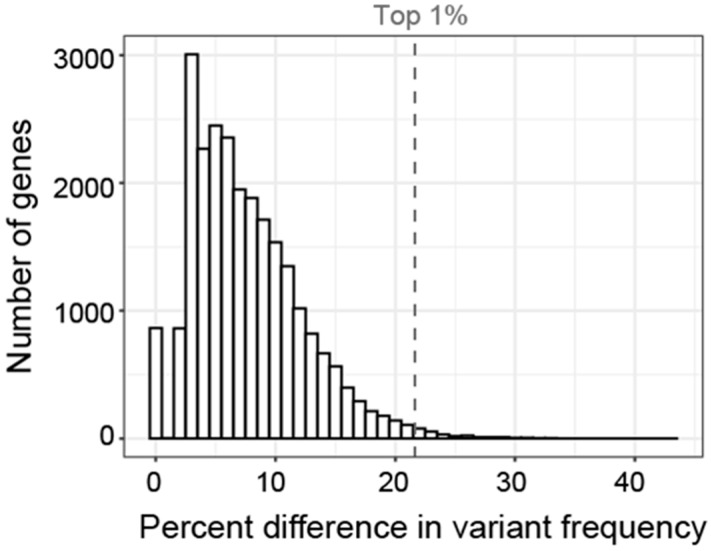
Histogram showing the distribution of the difference in GVF for each gene. Difference in GVF calculated as the maximum frequency minus the mean of the frequency in the other 5 subgroups. Only 1% of genes had a difference in GVF greater than 21% (gray dashed line).

To screen for relative enrichment of variants in a given subgroup, we calculated the average difference in GVF (Figure 9). For example, 69% of participants in the ‘Tactile and Auditory’ group had at least one variant in THSD7B. In contrast, other subgroups had GVFs ranging from 39% to 53% for an average of 47%. Thus, the average difference in GVF was 22% (4th highest magnitude of all genes) indicating variants in THSD7B were 22% more common in the ‘Tactile and Auditory’ group compared to other subgroups. In contrast, the mean allele frequency of these variants as listed in gnomAD is 0.12%.

It is not known what percent difference in GVF is clinically significant and our cohort is not powered to identify statistically significant correlations. In addition to evaluating the genes with the highest GVF in each subgroup, we considered a gene associated with each subgroup if the GVF was more than 2 standard deviations above or below the GVF for all other subgroups. We then used a two-sided t-test to compare the number of associated genes in our analysis with the number of genes meeting that criteria across 100 permutations of randomly assigning genes to each subgroup and found them to be significantly different (*p* < 0.05) suggesting our associations did not occur by chance. Further, we looked to see how often each of the associated genes was associated with any subgroup in the random permutations and for all of the genes identified as associated in our analysis, the chance of being associated with any particular subgroup was always less than 5%. To fully evaluate if these associations are causative, functional studies will be necessary.

For the three subgroups characterized by specific sensory patterns we found enrichment for variants in unique patterns of genes. The ‘Tactile and Auditory’ subgroup was enriched for variants in ABCA12, ADAMTS18, ELN, GRAMD1B, JPH3, OLA1, PAPPA, THSD7B and TMEFF2 (Table 4). The ‘Tactile and Auditory Hyposensitivity’ group was enriched for variants in KDM4C, MGMT, PKD1, SHANK1, and TNXB (Table 4). The ‘Taste and Auditory’ subgroup was enriched for variants in eighteen genes (Table S1), however, all of these genes negatively correlated meaning participants were less likely to have variants. The ‘Mixed’ group was enriched for variants in GRIN2B, HNRNPUL2, EPHB1, LRPPRC, SORL1, MEIS1, and SSH2 (Table 4). Interestingly, four of these seven genes are associated with a neurological disorder such as epileptic encephalopathy or Hereditary Spastic Paraplegia. The ‘Typically responding in all areas’ subgroup and the “globally atypically responding” subgroup were enriched for variants in a larger number of genes (Table S1).

## 3. Discussion

### 3.1. Sensory Features Can Be Used to Identify Unique Subgroups in ASD

The primary aim of this study was to identify sensory-based subgroups in ASD that potentially share underlying genetic mechanisms as defined by unique patterns of genes with high frequency of variants. Our results demonstrate that cluster analysis can be used to identify distinct subgroups with unique sensory phenotypes. Importantly, our analysis is unique from prior cluster analysis of sensory features through our use of hyper- and hypo-sensitivity specific identification. Quantitatively, K = 3 created the most specific clusters as measured by cluster density (Figure S1) consistent with sensory clusters identified in two prior studies [13,29]. In order to evaluate patterns of sensory differences, we looked at higher cluster numbers which, in addition to two subgroups categorized by involvement of many sensory processing differences and a group characterized by minimal differences in sensory areas, identified three subgroups with unique sensory patterns associated with variants in specific genes. Cluster analysis easily discriminated these sensory-based subgroups, proving feasibility for this type of analysis. Further, these results provide the first clinical phenotype—genotype evidence that shared sensory features in ASD may arise from shared underlying molecular mechanisms. Overall, these data support the idea that sensory features are strong candidates to guide patient classification in treatment trials.

Using sensory features to resolve heterogeneity in ASD has been proposed previously (reviewed in [20]) [13,30,31,32,33,34,35,36]. Most studies use parental surveys such as the SSP or the SSP-2. Notably, there is little consensus across studies other than a “very affected” group and a “minimally affected” group, both of which were also identified in our study. Specifically, six studies have previously reported cluster analysis using SSP data. The first two studies from the same group reported model-based cluster analysis in R using data from 54 and 30 participants respectively. The first analysis identified 3 clusters distinguished by scoring exclusively in taste/smell sensitivity and low energy/weak with participants being either typical in both, atypical in both, or atypical only in Taste/Smell [32]. The follow up study further subdivided into 5 clusters, but again was based primarily on taste/smell [33]. The same group followed up with a larger study (*N* = 228) in which they identified 4 clusters, one of which was again taste/smell sensitive [13]. The importance of taste/smell as a driver in sensory based clusters is consistent with the ‘Auditory and Taste Hyposensitivity’ specific cluster identified in this study. The next report of sensory based clusters using the SSP also used model-based cluster analysis in a cohort of 57 children but only identified 3 clusters characterized by mostly typical responses, mostly atypical responses and “other” consistent with the three cluster groupings identified in our cohort suggesting further cluster sub-identification could have been applied to that cohort [29]. Finally, the two most recent cluster analysis of SSP data using 400 children ages 3–6 and 599 children ages 1–21 years, respectively, did not report sensory modality specific clusters characterizing them as sensorimotor, selective-complex, perceptive-adaptive and vigilant-engaged [36] and sensory adaptive, generalized sensory differences, taste and smell sensitivity, underresponsive and sensory seeking and Movement and Low Energy/Weakness [21]. It would be very interesting to compare our modality specific analysis to these clusters or to consider these terminologies in our cohort, but given the different goals of the study, it is challenging to directly compare them. We chose to focus on hyper- and hypo-sensitivity because these qualities are more easily quantified using direct sensory testing in both patients and animal models. Future analysis using surveys targeting hyper- and hypo-sensitivity may help to reduce noise in patient datasets and allow direct comparison between cluster analysis using these various models.

### 3.2. Genes Enriched within Each Sensory Subgroup Are Relevant to ASD

Identifying less heterogeneous subgroups within ASD has been proposed as a potential key to developing targeted treatments [7,37]. Subgrouping using various clinical features has been done [38,39] but it has been increasingly recognized that the genetic signature of each subgroup will be important [6,40]. While sensory based subgroups in ASD have been proposed (reviewed in [20], largest cohort to date [21]), this study is the first to investigate these sensory based subgroups for enrichment of genetic variants. While our study was not powered to identify significant correlations with variants in specific genes, our results suggest that correlating sensory phenotypes with genetic variants will be an important and fruitful area of future study. Here, we demonstrate a model for how this analysis can be done by collapsing all variants for each gene, which has been done previously in genome-wide-association studies in Parkinson’s disease [28].

Interestingly, based on measures using the SSP, individuals with Phelan-McDermid Syndrome are more likely to have taste/smell and visual/auditory sensitivity compared to individuals with idiopathic ASD but no differences in tactile sensitivity or seeking, suggesting specific genetic mechanisms can result in distinct sensory differences [15]. However, which genes are important for each specific sensory difference is not known. To address this gap, it will be important to carefully characterize patterns of sensory processing differences in monogenetic neurodevelopmental disorders.

Several genes that were uniquely enriched in specific subgroups are relevant to ASD. For example, we found that the Tactile and Auditory hyposensitivity subgroup (subgroup 4), had increased variants in *PKD1*. *PKD1* is a protein kinase expressed in rat and mouse dorsal root ganglia [41] and Merkel cells [42] consistent with a role in tactile sensory processing. *PKD1* is associated with polycystic kidney disease, some clinical forms of which present with hearing impairment, although to our knowledge auditory sensitivity has not been tested in the *PKD1* knockout mouse. This same subgroup exhibited decreased variants in *SHANK1*. The *SHANK1* knockout mouse is a model for ASD [43,44]. *SHANK1* knockout mice do not exhibit deficits in discrimination of dissimilar odors (banana vs. almond) or auditory startle [45] although it is less clear how to interpret a decrease in variants within a subgroup. Some of the identified genes have been previously associated with neurological disorders. For example, the Mixed subgroup (subgroup 6) had increased variants in *GRIN2B* (Table 4). *GRIN2B* is a NMDA receptor subunit associated with developmental and epileptic encephalopathy [46]. Thus, all of these genes are strong candidates to identify a shared molecular mechanism contributing to the specific sensory phenotype for that subgroup. Future animal model studies investigating hyper- and hypo-sensitivity in mouse models with the genetic variants identified in these subgroups will be necessary to elucidate the specific mechanistic connection for those subgroups.

### 3.3. Limitations

This study has several limitations. Most importantly, it is a retrospective study which greatly limited the available data. For example, while MSSNG has whole genome sequences for over 10,000 individuals with ASD, Short Sensory Profile Responses with whole genome sequencing were available for only 364 individuals. Thus, the final number of participants in each cluster was relatively small and this study was not powered to identify statistically significant correlations. Obtaining whole genome sequencing and sensory behaviors from large numbers of children is logistically challenging requiring significant funding. Importantly, even in this small cohort, characterization of subgroups identified specific patterns supporting the use of sensory features as a biomarker to correlate with genetic variants and suggesting sensory phenotyping should be a priority in future multi-site efforts. We hope that future studies will prioritize collection of sensory features to allow expansion of this type of analysis.

A second limitation is that this sample set included children ages 23 months to 21 years which is beyond the SSP recommended age range of 3–14 years. We did not observe any difference in mean age across clusters (Table 3) and sensory features have been shown to be stable over time in ASD [47], but it is possible that sensory sensitivities in these extreme ages were not well characterized. Importantly, the SSP evaluates sensory behaviors in seven domains evaluating ‘sensory processing patterns’ and ‘self-regulation strategies’ which is a more global or holistic view of sensory processing [48,49]. While the SSP is well validated and is very useful for clinically understanding the impact of sensory changes in ASD, it does not directly test for hyper- and hypo-sensitivity which are potentially easier to link to circuit mechanisms. We addressed this limitation by using a subset of responses focusing on hyper- and hypo-sensitivity (Table 2). However, not all sensory modalities were equally represented. Thus, future studies should use direct measure of sensory ability and more targeted questionnaires to validate the subgroups identified.

Finally, this study collapsed all variants with a CADD score greater than 15 and thus included potentially non-deleterious variants but did not include frameshifts or deletions. Further, no distinction was made between heterozygous or homozygous variants. This approach was taken in order to identify broad genetic patterns within the identified subgroups. As variant frequency was compared between subgroups, even if the individual variants themselves are not predicted to be deleterious, the difference between subgroups is still relevant. However, functional studies will be necessary to test if the identified genes are causative of the sensory changes.

## 4. Materials and Methods

The aim of this study was to identify subgroups of individuals with ASD based on their sensory phenotypes and to determine if these subgroups shared underlying genetic mechanisms as defined by unique patterns of genes with high frequency of variants. Towards this aim, this study utilized Short Sensory Profile (SSP) data and whole genome sequencing (WGS) results for 378 participants, of which 80% (303/378) were male, obtained from the Autism Speaks’ MSSNG Database. Age at time of testing calculated using month and year of birth and test date was available for 375 participants ranging from 23 months to 21 years (mean ± std, 9.87 ± 4.67). 72% (272/378) of participants had a European predicted ancestry. Other predicted ancestries included East Asian (4.2%), Other (10%), ADMIXED (5.3%), South Asian (4%), African (3.2%) and American (1.3%). Full scale IQ testing based on most recent edition of Wechsler, Stanford-Binet or Mullen was available for 226 participants with a mean and standard deviation of 79.8 ± 27.7. The MSSNG database includes a normalized adaptive behavior standard score calculated from the Adaptive Behavior Assessment Scale (ABAS) or the Vineland and a socialization standard score from the ABAS. These data were available for 374 and 373 participants, respectively, with mean scores of 68.1 ± 14.9 and 70.4 ± 14.1 respectively. Demographic features were not statistically significantly different across subgroups (Table 3).

### 4.1. Procedures

The SSP [27] is a 38-question parent survey that quantifies frequency of sensory behaviors using a 5-point likert scale (1 = always, 5 = never). SSP data have previously been used to assess sensory features in many neurodevelopmental disorders [50,51,52,53] including ASD [23,24,25,26]. Questions are divided into seven sections termed sensory areas: Tactile Sensitivity, Taste/Smell Sensitivity, Movement Sensitivity, Underresponsive/Seeks Sensation, Auditory Filtering, Low Energy/Weak, and Visual/Auditory Sensitivity. Scores are summed in each area and compared to standardized scores for typically developing children. Respondents are categorized as typically responding, probably different, or definitely different.

The SSP quantifies the frequency of sensory behaviors which are challenging to directly measure, but hyper- and hypo-sensitivity can be directly measured in people and animals by testing detection thresholds. Hyper-sensitivity is an increased response to a sensory stimulus or ability to detect lower intensity stimuli whereas hypo-sensitivity is an absent, delayed or decreased response to sensory stimulation. Therefore, identification of hyper- and hypo- sensitivity clusters would be clinically useful. In order to more directly assay hyper- and hypo-sensitivity, we extracted questions that asked specifically about hyper- or hypo-sensitivity in each of the four main sensory domains mentioned in the SSP using comparison to questions in other sensory survey tools and best clinical judgement with input from sensory experts (Table 2). Two of the sensory areas in the SSP evaluate features outside the five main sensory domains of hearing, vision, touch, taste, and smell. For example, the Movement Sensitivity category asks how often the child “fears falling or heights.” These questions were not included in the hyper- and hypo-sensitivity sub-analysis. Similarly, some questions regarding primary senses cannot be extrapolated to hyper- or hypo-sensitivity, such as “Has difficulty standing in line or close to other people.” Scores for the subset of questions within each sensory domain were summed and assigned as typically responsive or hyper/hypo responsive using adjusted cut-offs based on the SSP validated data [27]. Cluster analyses were performed using both the full SSP and this subset of questions.

### 4.2. Data Analysis

Histogram visualization of the SSP responses in each of the seven sensory areas and the four sensory domains allowed evaluation of the range of responses for all 378 participants. One participant was excluded from further analysis due to absence of variance in the responses preventing inclusion in correlation-based clustering methods. Next, we performed two separate cluster analyses on SSP responses from 377 individuals using the responses to all 38 questions or the subset of responses focused on hyper-and hypo-sensitivity (Table 2). The cluster analyses were done using K-mean clustering on bootstrap samples and then using consensus clusters from the bootstraps. The median distribution of consensus clusters for K = 3–10 from 38Question were compared to select the optimal cluster number (Figure S1). We then cross-compared subgroups identified by each clustering method to clarify the characteristics associated with each cluster. We assessed the similarity of compared subgroups with rand index.

### 4.3. Variant Extraction and Statistical Analysis

WGS results within the MSSNG database are stored in Google BigQuery. All single nucleotide variants (SNV) that were annotated to a gene and had a minimum Combined Annotation Dependent Depletion (CADD) [54] score of 15 were extracted from this database. A CADD score of 10 represents the top 10% most deleterious variants while a CADD score of 20 represents the top 1%. We selected an intermediate CADD threshold of 15 which likely included both rare and common variants. We controlled for effect of ancestry by confirming that predicted ancestry distribution was not statistically different between clusters. This list was then used to call variants from each participant. Called variants were aggregated at a gene level for each participant meaning a variant was marked observed if at least one variant annotated to the gene was found regardless of the variant type. This method identified 24,896 genes for which at least one participant had a variant. As an exploratory analysis, we calculated the percent of patients in each subgroup who had variants with a CADD score of 15 or greater in each gene and we call this gene variant frequency (GVF). By collapsing across variants within genes, we aimed to be hypothesis generating rather than demonstrating variant specific correlations [28]. We then identified the ten genes with the highest GVF for each subgroup and the genes that were uniquely enriched for variants within each subgroup to identify candidate genes for future analysis.

## 5. Conclusions

Using Short Sensory Profile (SSP) data from 377 individuals with idiopathic ASD obtained through the MSSNG database, we identified six sensory—based subgroups. These subgroups exhibited high impairment across multiple domains, absence of hyper- or hypo-sensitivity, or discreet combinations of sensory features. Further, each of these subgroups was enriched with a unique set of genetic variants with higher enrichment for ASD related genes. We conclude that sensory features can be used to identify subgroups within ASD with shared patterns of genetic variants. These results represent the first step towards identifying mechanistically linked subgroups important for targeted drug development and evaluating efficacy in therapeutic trials.

## Figures and Tables

**Table 1:** 10 most common combinations of responses for the 7 sensory areas.

Description	*N*
Definitely or probably different in all areas	54
Definitely or probably different in all areas EXCEPT Low Energy/Weak	15
Definitely or probably different in all areas EXCEPT Taste/Smell	14
Definitely or probably different in all areas EXCEPT Movement	13
Definitely or probably different in: Visual/Auditory, Taste/Smell, Under-responsiveness/Seeks Sensation, Tactile and Auditory Filtering	13
Definitely or probably different in: Under-responsiveness/Seeks Sensation, and Auditory Filtering	13
Definitely or probably different in: Under-responsiveness/Seeks Sensation, Low Energy/Weak, and Auditory Filtering	11
Definitely or probably different in: Under-responsiveness/Seeks Sensation, Tactile, and Auditory Filtering	10
Definitely or probably different just in Auditory Filtering	10
Typical performance in all areas	9

**Table 2:** Subset of questions from the SSP used to evaluate hyper- and hypo- sensitivity.

Sensory Domain	SSP Question Number and Text	
Vision Hypersensitivity	36	Is bothered by bright lights after others have adapted to the light
	38	Covers eyes or squints to protect eyes from light
Auditory Hypersensitivity	34	Responds negatively to unexpected or loud noises (for example, cries or hides at noise from vacuum cleaner, dog barking or hair dryer)
	35	Holds hands over ears to protect ears from sound
Auditory Hyposensitivity	23	Appears to not hear what you say (for example, does not “tune-in” to what you say, appears to ignore you)
	26	Doesn’t respond when name is called but you know the child’s hearing is OK
Tactile Hypersensitivity	1	Expresses distress during grooming (for example, fights or cries during haircutting, face washing, fingernail cutting)
	3	Avoids going barefoot, especially in sand or grass
	4	Reacts aggressively or emotionally to touch
	5	Withdrawals from splashing water
	7	Rubs or scratches out a spot that has been touched
Tactile Hyposensitivity	18	Touches people and objects
	19	Doesn’t seem to notice when face or hands are messy
Taste Hypersensitivity	8	Avoids certain tastes or food smells that are typically part of children’s diet
	11	Picky eater, especially regarding food textures

**Table 3:** Participant features for each subgroup.

Subgroup	Total Number	Male N (%)	European Ancestry N (%)	Mean Age Years	Mean Adaptive Behavior Score	Mean Socialization Score	Full Scale IQ Mean (N)
**1**	53	45 (84.9)	39 (73.6)	10.3	64	67	89 (33)
**2**	72	58 (80.5)	48 (66.7)	10.0	73	76	80 (39)
**3**	63	51 (80.9)	46 (73.0)	8.0	66	67	80 (45)
**4**	62	55 (88.7)	42 (67.7)	9.8	66	70	73 (36)
**5**	67	45 (67.1)	49 (73.1)	9.1	69	72	80 (44)
**6**	60	48 (80.0)	47 (78.3)	12.1	67	69	77 (29)
**Total**	377	302 (80.1)	272 (72.0)	9.87	68	70	80 (226)

**Table 4:** Genes with uniquely high variant frequency in subgroups 3, 4 and 6.

Subgroup		Gene	Variant Frequency	Difference	Gene Card Summary	Gene Ontology
Tactile and Auditory	3	THSD7B	0.69	0.22	Glycosylation	Protein binding
	3	GRAMD1B	0.45	0.21	Cholesterol transport	Phosphatidylserine, lipid, cholesterol and phosphatidic acid binding; intermembrane cholesterol transfer activity
	3	ABCA12	0.45	0.20	Transporter	signaling receptor binding and ATPase activity, coupled to transmembrane movement of substances
	3	ADAMTS18	0.42	0.17	Metalloproteinase	peptidase activity and metalloendopeptidase activity
	3	ELN	0.23	0.15	Extracellular matrix	extracellular matrix structural constituent and extracellular matrix constituent conferring elasticity
	3	JPH3	0.29	0.15	Junctional Complexes	calcium-release channel activity
	3	TMEFF2	0.47	0.15	Oncogene	Protein binding
	3	OLA1	0.24	−0.15	GTPase protein family	GTP binding and ribosome binding
	3	PAPPA	0.26	−0.20	Metalloproteinase	metalloendopeptidase activity and endopeptidase activity
Tactile and Auditory Hyposensitivity	4	PKD1	0.41	0.16	Glycoprotein	protein kinase binding and protein domain specific binding
	4	KDM4C	0.48	0.15	Demethylase	enzyme binding and dioxygenase activity
	4	MGMT	0.29	−0.14	Transfer of methyl groups	calcium ion binding and damaged DNA binding
	4	TNXB	0.16	−0.16	Extracellular matrix	heparin binding and collagen binding
	4	SHANK1	0.17	−0.19	Scaffold proteins	Identical protein binding, Protein C-terminus binding
Mixed	6	GRIN2B	0.553571	0.159151	NMDA-R subunit	calcium channel activity and ionotropic glutamate receptor activity
	6	HNRNPUL2	0.214286	0.140732	Nuclear Ribonucleoprotein	kinase activity
	6	EPHB1	0.303571	−0.1433	Receptor Tyrosine Kinase	transferase activity, transferring phosphorus-containing groups and protein tyrosine kinase activity
	6	LRPPRC	0.142857	−0.14576	Mitochondrial	ubiquitin protein ligase binding
	6	SORL1	0.125	−0.14501	Vacuolar sorting protein LDL receptor	transmembrane signaling receptor activity and low-density lipoprotein particle binding
	6	MEIS1	0.428571	−0.16274	Homeobox	sequence-specific DNA binding and chromatin binding
	6	SSH2	0.160714	−0.16829	Tyrosine phosphatase	actin binding and protein tyrosine phosphatase activity

## Data Availability

The datasets analyzed during the current study are available in the MSSNG repository, https://research.mss.ng (accessed from December 2019 to September 2021). Restrictions apply to the availability of these data, which were used under license for the current study, and so are not publicly available. Data are however available from Autism Speaks following appropriate request for access.

## Supplementary Materials

The following supporting information can be downloaded at: https://www.mdpi.com/article/10.3390/ijms232113030/s1.
