# Supplementary material for: Cluster Analysis of Short Sensory Profile Data Reveals Sensory-Based Subgroups in Autism Spectrum Disorder
Source: Int J Mol Sci. 2022 Oct 27;23(21):13030. doi: 10.3390/ijms232113030 (PMC9655407; doi:10.3390/ijms232113030)

Supplementary Table S1: Genes associated with groups 1, 2 and 5

Group	Gene	GVF (%)	Difference
1	AHDC1	63	27
1	AJAP1	37	18
1	ASPH	33	18
1	ATF2	23	15
1	BBOX1	27	14
1	BCL11B	50	18
1	BTBD9	56	16
1	CCDC30	31	18
1	CFAP58	33	15
1	COL25A1	58	22
1	DCST1	27	15
1	DPYSL2	40	23
1	ENPEP	27	14
1	ERBB4	81	17
1	FAM161A	29	16
1	FBXW11	33	19
1	FNIP1	31	15
1	GCOM1	35	14
1	GIGYF2	40	18
1	HERC1	46	17
1	INHBE	17	15
1	IPO7	35	18
1	KLF5	37	17
1	LINC00499	35	17
1	LINC01697	29	15
1	LNX1	27	15
1	LOC101929950	33	16
1	LRIG1	42	19
1	MIR548XHG	35	19
1	MPPED2	56	18
1	MSRA	50	18
1	MTA3	35	15
1	MYO6	27	15
1	NCAM2	42	18
1	NWD2	35	18
1	OR10T2	19	15
1	PCBP2	44	23
1	PCDHA13	33	14
1	PCM1	38	18
1	PDE4DIP	44	17
1	PKDCC	31	17
1	POLD1	17	15
1	PTPRJ	35	19
1	RALBP1	33	22
1	SALL2	31	16
1	SCARF2	31	17
1	SGIP1	37	15
1	SH3PXD2A	46	19
1	SIGLEC1	29	15
1	SIM1	37	17
1	SNX24	38	15

Group	Gene	GVF (%)	Difference
1	SPEG	42	18
1	TRPM7	33	17
1	TSHZ3	56	20
1	TSPEAR	52	16
1	VPS13B	60	17
1	WAPL	31	14
1	ZBTB4	44	18

Supplementary Table S1 continued

Group	Gene	GVF (%)	Difference
2	ADARB2	42	16
2	ANKRD45	19	15
2	BACH2	51	16
2	C5orf66	58	22
2	CCDC85A	37	17
2	FAM222B	27	15
2	FOXP2	61	17
2	FREM2	37	17
2	HAO1	25	17
2	IGF1R	49	14
2	IGSF3	25	16
2	JAZF1	54	14
2	KRT17P1	21	15
2	LINC01934	43	18
2	LINGO1	46	18
2	MACROD1	51	17
2	MBD6	30	15
2	MIA2	30	14
2	MRPS6	37	17
2	NRG3	60	21
2	PALD1	24	15
2	PTPRS	42	16
2	SETD5	42	16
2	SKAP1	76	21
2	SMG1P2	30	17
2	TAOK3	43	19
2	TLL1	28	15
2	TSHZ2	73	17
2	USHBP1	21	15
2	WDFY2	21	17
2	ZNF462	55	15

Group	Gene	GVF (%)	Difference
5	C11orf49	13	-18
5	CDH13	55	-17
5	CHD7	20	-18
5	CTNNBL1	16	-17
5	ETS1	16	-18
5	EYS	28	-21
5	FOXP2	33	-17
5	GRIK4	23	-19
5	KIAA1217	28	-16
5	KIRREL1	13	-18
5	MBNL2	14	-15
5	MSI2	53	-17
5	SEMA5B	13	-14
5	SLC10A7	16	-17
5	ST6GALNAC5	14	-16
5	TSNAX-DISC1	13	-15
5	WDFY4	22	-14
5	ZNF827	20	-17

Supplementary Figure S1: Distribution of consensus for K= 3-10

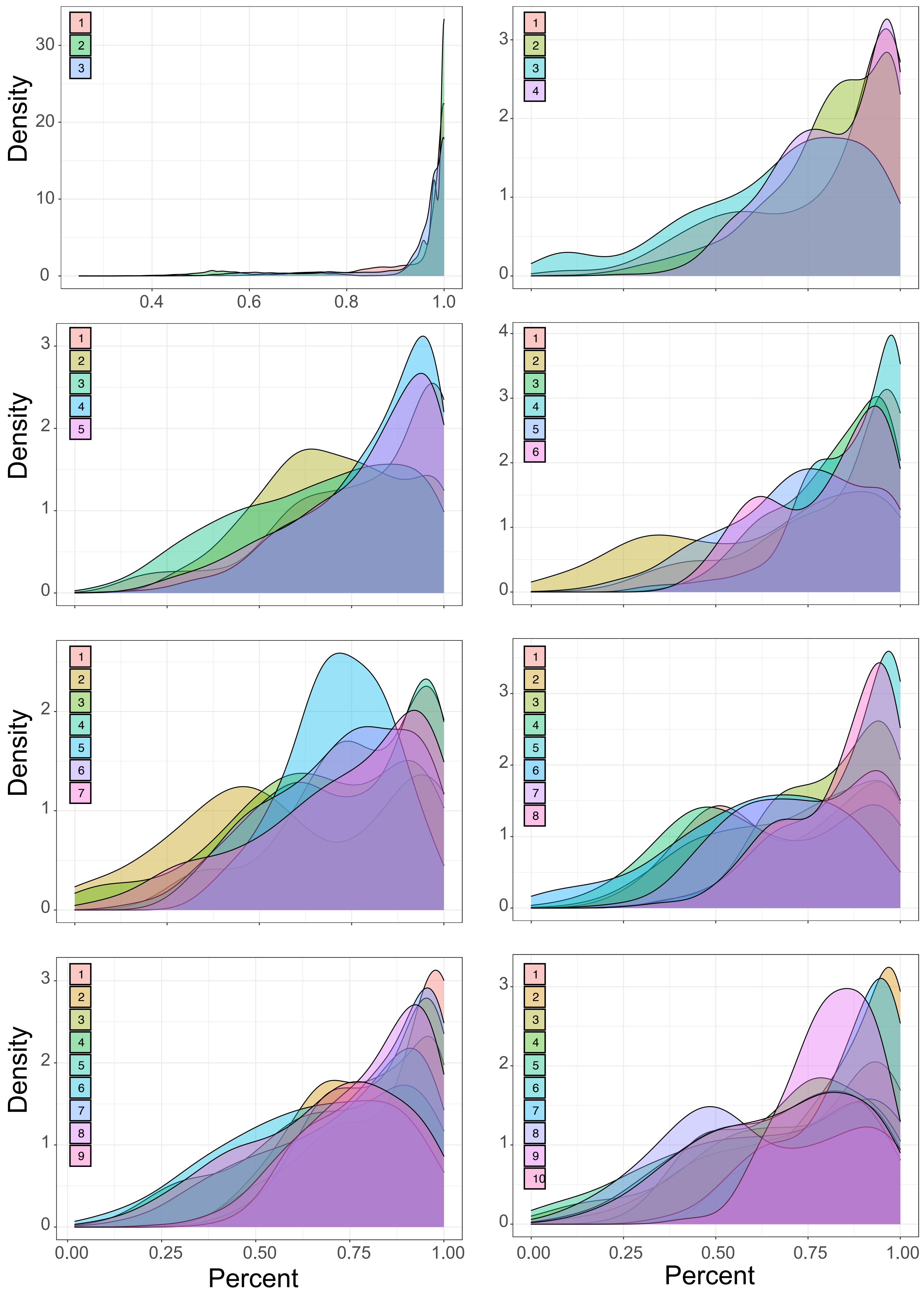

Supplement: Supplementary file 1 [file ijms-23-13030-s001.zip › ijms-1833616-supplementary.pdf]
